# Distance to health services and treatment-seeking for depressive symptoms in rural India: a repeated cross-sectional study

**DOI:** 10.1017/S204579601900088X

**Published:** 2020-01-13

**Authors:** T. Roberts, S. Shiode, C. Grundy, V. Patel, R. Shidhaye, S. D. Rathod

**Affiliations:** 1 Health Service & Population Research Department, Institute of Psychiatry, Psychology & Neuroscience, King's College London, London, UK; 2 Department of Geography, Birkbeck University of London, London, UK; 3 MRC Tropical Epidemiology Group, Epidemiology & Population Health Faculty, London School of Hygiene & Tropical Medicine, London, UK; 4 Department of Global Health and Social Medicine, Harvard Medical School, Boston, MA, USA; 5 Centre for Mental Health, Public Health Foundation of India, New Delhi, India; 6 Care and Public Health Research Institute, Maastricht University, Maastricht, Netherlands; 7 Department of Population Health, Epidemiology & Population Health Faculty, London School of Hygiene & Tropical Medicine, London, UK

**Keywords:** Depression, health service research, primary care, minority issues, cross cultural psychiatry

## Abstract

**Aims:**

Research from high-income countries has implicated travel distance to mental health services as an important factor influencing treatment-seeking for mental disorders. This study aimed to test the extent to which travel distance to the nearest depression treatment provider is associated with treatment-seeking for depression in rural India.

**Methods:**

We used data from a population-based survey of adults with probable depression (*n* = 568), and calculated travel distance from households to the nearest public depression treatment provider with network analysis using Geographic Information Systems (GIS). We tested the association between travel distance to the nearest public depression treatment provider and 12 month self-reported use of services for depression.

**Results:**

We found no association between travel distance and the probability of seeking treatment for depression (OR 1.00, 95% CI 0.98–1.02, *p* = 0.78). Those living in the immediate vicinity of public depression treatment providers were just as unlikely to seek treatment as those living 20 km or more away by road. There was evidence of interaction effects by caste, employment status and perceived need for health care, but these effect sizes were generally small.

**Conclusions:**

Geographic accessibility – as measured by travel distance – is not the primary barrier to seeking treatment for depression in rural India. Reducing travel distance to public mental health services will not of itself reduce the depression treatment gap for depression, at least in this setting, and decisions about the best platform to deliver mental health services should not be made on this basis.

## Background

### Depression treatment gap

According to the World Health Organization (WHO), depression affects 4.4% of the world's population (WHO, 2017), but less than half are estimated to receive treatment (Kohn *et al*., 2004). In India, 1 in 20 people meets criteria for depression but fewer than 15% of these report seeking treatment (Gururaj *et al*., 2016). There are global efforts underway to reduce this ‘treatment gap’ by integrating mental health care into primary care services, as exemplified by the five-country Programme to Improve Mental Health Care (PRIME) (Lund *et al*., 2012).

### Access to care and geographic accessibility

The geographic accessibility of health services is implicated in most major models of access to care (Aday and Andersen, 1974; Penchansky and Thomas, 1981; Peters *et al*., 2008; De Silva *et al*., 2014). Reducing distance to the nearest mental health service through strategies such as decentralisation and integration is therefore expected to lead to increases in service uptake; a phenomenon known as ‘Jarvis’ Law’ (Hunter and Shannon, 1985).

In India, greater distance to facilities has been linked to reduced treatment-seeking for general and maternal health needs, particularly affecting disadvantaged groups such as scheduled tribes and women (Sawhney, 1993; Kumar *et al*., 1997; Vissandjée *et al*., 1997; Shariff and Singh, 2002; Ager and Pepper, 2005; Kumar *et al*., 2014). To our knowledge, no studies have tested this association for mental disorders in an Indian context.

### Mental health systems in India

India has a great variety of healing systems, including allopathic (biomedical) services, indigenous forms of health care (including Ayurveda, yoga, naturopathy, Unani, Siddha, homoeopathy and local systems of medicine), and spiritual or religious healing (Halliburton, 2004). Patients' explanatory models of mental illness may align more closely with those of traditional or religious practitioners than biomedical models (Wilcox *et al*., 2007) but the parallel use of multiple systems is common (Albert *et al*., 2015; Shankar, 2015).

Private services have also become increasingly dominant in the Indian health system (De Costa and Johannson, 2011), with 80% of outpatient consultations now taking place in the private sector (Selvaraj and Karan 2009; Kumar *et al*., 2011). Much of this sector is composed of small-scale practitioners with little or no formal training (De Costa and Diwan, 2007; Ranga and Panda, 2016), many of whom dispense psychopharmacological treatment (Ecks and Basu, 2009, 2014).

Nonetheless, the existence of traditional and informal services is frequently ignored in discourse on mental health care in India (Quack, 2012). Both Indian mental health policy (Ministry of Health & Family Welfare, 2014) and WHO-recommended strategies to expand access to mental health treatment (WHO, 2008) focus on public, allopathic services.

### PRIME

Through PRIME, a mental health care plan (MHCP) was implemented in 2014 in Sehore district, Madhya Pradesh, in partnership with the state Ministry of Health (Shidhaye *et al*., 2016*b*). The MHCP aimed to reduce the treatment gap for priority mental disorders by integrating services into public primary care facilities, thus making them more geographically accessible to the rural population. Increased accessibility of public, allopathic mental health services (the target of PRIME) was expected to reduce the treatment gap for depression (a key goal of PRIME). While this expectation arguably overlooks the great variety of care systems in the Indian context, it mirrors current accepted wisdom in global mental health that the treatment gap reflects limited access to formal mental health care. We therefore set out to test this hypothesis empirically, to inform future initiatives to reduce the depression treatment gap.

### Objectives

This study aimed to:

1. Compare travel distance by road from the households of individuals with depression to the nearest public depression treatment provider, before and after implementation of the MHCP.
2. Measure the association between travel distance to the nearest public depression treatment provider and the probability of treatment-seeking for probable depression in rural India.
3. Assess whether this association varies by gender, caste, symptom severity, socio-economic status (as measured by housing type, employment status, land ownership and education level) and perceived need for healthcare.

## Methods

### Setting

**Fig. 1.**
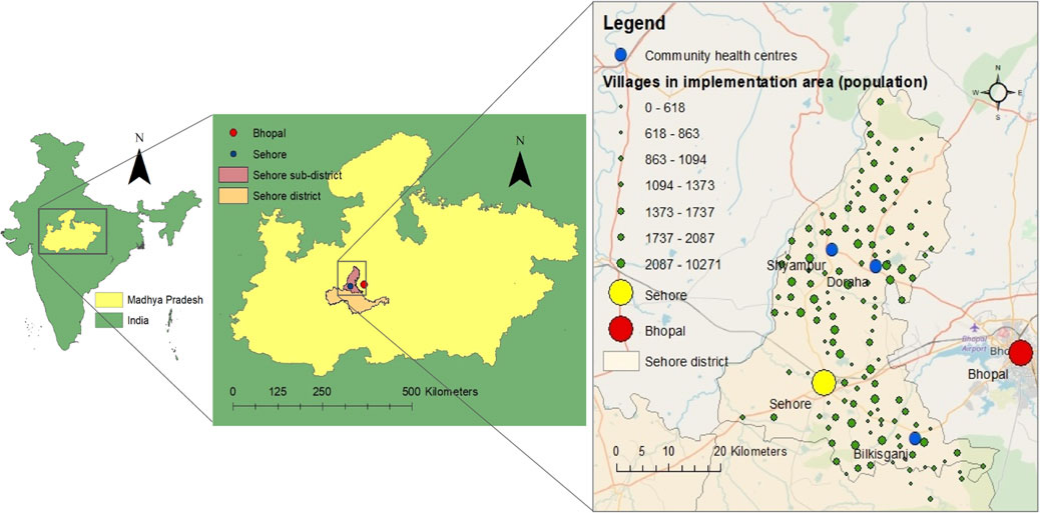
Map of study area, showing location of villages within implementation area, community health centres, and towns/cities where public depression treatment services were previously available (Bhopal/Sehore).

Sehore sub-district, within Sehore district, Madhya Pradesh (Fig. 1) is 74% rural, with a population of 427 432. Fewer than 4% own cars and 34% own scooters/motorcycles, with lower proportions among rural residents (Office of the Registrar General and Census Commissioner, 2011). Prior to MHCP implementation there were two public mental health specialists serving a district population of 1.3 million (Hanlon *et al*., 2014).

The study area (Shidhaye *et al*., 2015), MHCP (Shidhaye *et al*., 2016*b*) and evaluation plan (De Silva *et al*., 2016) have been previously described. Psychological interventions for depression were delivered by case managers and pharmacological treatments prescribed by medical officers at Community Health Centres (CHCs). Case managers conducted community case-finding and screened patients in CHCs. Some community awareness activities were conducted, such as meetings and film screenings. The term ‘implementation area’ refers to those villages where MHCP activities were fully implemented (see Fig. 1).

### Data collection

As part of the PRIME evaluation, we carried out a population-based community survey with two rounds, with the primary aim of measuring a change in the proportion of people with depression and alcohol use disorders who sought treatment. The data collection methods and sampling strategy have been described elsewhere (De Silva *et al*., 2016; Rathod *et al*., 2016). Data collection for the first round took place prior to MHCP implementation, in two waves (May–June 2013, January–March 2014). The second round was conducted after MHCP implementation (October–December 2016). Inclusion criteria were: aged ⩾18, fluency in spoken Hindi, residency in selected households, willingness to provide informed consent and absence of cognitive impairments that would preclude informed consent or ability to participate.

This secondary analysis of the survey data considered residents of the MHCP implementation area with probable depression. Since there was no difference in the proportion of people who sought treatment for depression between rounds (Shidhaye *et al*., 2019), we pooled data from both rounds for analysis. Across both rounds, 6201 adults were recruited. A total of 6134 (98.9%) consented to participate. Of these, 4297 resided within the implementation area, of whom 568 had probable depression (round 1: 289, round 2: 279).

Questionnaires were administered orally, in Hindi, by trained local fieldworkers. Fieldworkers recorded participant responses using a questionnaire application programmed on Android tablet devices, which also recorded the interview location's GPS coordinates.

### Measures

The screening tools and other measures are described in detail elsewhere (Rathod *et al*., 2016). Briefly, we measured current depression symptoms using the Patient Health Questionnaire, 9-item version (PHQ-9), using the standard cut-off point of ⩾10 (Manea *et al*., 2012). In an international meta-analysis, the PHQ-9 had a pooled sensitivity of 0.77 (95% CI 0.66–0.85) and specificity of 0.85 (95% CI 0.79–0.90) to detect major depressive disorder with this criterion (Manea *et al*., 2015). The main outcome of interest was treatment-seeking for depression symptoms, measured by asking: ‘Did you seek any treatment for these problems at any time in the past 12 months?’ Participants who responded affirmatively were asked to specify from whom they had sought treatment. These were divided into formal providers (generalist and specialist health workers, in the public or private sector) and complementary providers (traditional and alternative healers). Case managers, who were available in round 2 only, were categorised as formal providers. Additionally, we collected data on socio-demographic characteristics and barriers to health care use (Rathod *et al*., 2016).

#### Geographic measures

Household coordinates were missing for 62.8% of round 1 data and 17.6% of round 2 data. In these cases, we substituted coordinates for the village centre from India Place Finder (Mizushima Laboratory, 2013). These are based on geographic information from the 2001 Census of India, which we cross-referenced with mean GPS coordinates for households in the village. For households with GPS coordinates, the mean difference between households and their respective village centres was 935 metres (s.d. = 746 m).

The primary distance measure used was the shortest distance by road to the nearest public depression treatment provider (referred to as ‘travel distance’), calculated using network analysis in ArcGIS 10.5 (Esri, 2011). This is a recommended measure of geographic accessibility in contexts where most travel is vehicular (Delamater *et al*., 2012), as in 77.8% of recent health care visits reported by participants. We defined the nearest public depression treatment provider as the nearest of: Sehore city or Bhopal city only (rounds 1 and 2), plus any of the three CHCs (round 2 only). We used Open Street Maps (© OSM contributors) road network data to calculate travel distance, after cleaning these data to ensure connectivity. Since some households were located at a distance from the nearest road, we added straight line distances to the nearest road to estimate total travel distance.

### Analysis strategy

We first described the socio-demographic characteristics of the sub-sample, stratified by travel distance (0 < 5 km, 5 < 10 km, 10 < 20 km, ⩾20 km).

We then compared the median travel distance from cases to a public depression treatment provider by round using the Mann–Whitney test.

Next we estimated the change in odds of treatment-seeking associated with travel distance (in kilometres) to the nearest public depression treatment provider. We considered the following covariates as potential confounders in a logistic regression model; age, education level, gender, marital status, economic status (using housing type and employment status as proxy measures), symptom severity, disability, perceived need for health care, survey round and 12-month exposure to mental health communications. We excluded covariates from the final model after checking for collinearity with variance inflation factors and a correlation matrix of all variables. Regression analyses were repeated using two alternative outcomes: (a) any depression treatment and (b) treatment from the formal health sector only.

Next we used the final regression model to test for interaction effects. We hypothesised that the effect size would be larger for women and disadvantaged castes, those with milder symptoms, individuals with lower socio-economic status and those with a perceived need for health care, based on previous Indian and international literature. Stratum-specific effects are presented when a Wald test for all interaction terms had *p* < 0.10.

With the exception of counts, all figures were adjusted for the multi-stage sampling design and village-level clustering. Stata 14.2 (StataCorp, 2015) was used to conduct all analyses.

### Ethics

All participants were provided with an information sheet in Hindi, which was read aloud if required. After any questions were answered, they indicated informed consent with a signature or thumb print. The original survey, including the collection and analysis of GPS coordinates as part of the PRIME evaluation, was approved by the institutional review boards of Sangath, Goa, India; the Indian Council of Medical Research, New Delhi, India; WHO, Geneva, Switzerland and the University of Cape Town, Cape Town, South Africa. The current analyses form part of the work that was approved by these committees. Ethical approval for these analyses was additionally provided by London School of Hygiene & Tropical Medicine, London, UK (LSHTM Ethics Ref: 10439).

## Results

### Sample characteristics, by distance

Table 1 shows the characteristics of adults with probable depression, stratified by travel distance. A total of 69.6% of participants living <5 km from the nearest depression treatment provider were female, compared to 49.9% of those living more than 20 km away (*p* = 0.08). As shown in the table, the following sample characteristics varied by travel distance to the nearest facility: employment status; land ownership and religion.

**Table 1.** Demographic and health-related characteristics of adults with probable depression by travel distance to the nearest public health facility offering depression services, Sehore sub-district, Madhya Pradesh, India, 2013–2016

	0–5 km (*n* = 59, 8.5%)	5–10 km (*n* = 121, 18.7%)	10–20 km (*n* = 150, 24.5%)	20–123 km (*n* = 238, 48.3%)	Total (*n* = 568)	*p*-Value
Gender, %						
Female	69.6	60.5	51.0	49.9	53.8	0.08
Age groups (years), %						
18–29	22.0	20.8	17.5	15.5	17.5	0.85
30–49	41.0	42.5	45.1	44.7	44.1	
50–90	37.1	36.7	37.4	39.9	38.4	
Educational attainment, %						
Less than primary	79.9	77.0	70.7	73.7	74.1	0.20
Primary	18.6	21.2	22.2	23.6	22.4	
Secondary or more	1.5	1.8	7.1	2.7	3.5	
Employment status, %						
Unemployed	0.0	2.0	5.1	5.4	4.2	<0.01
Productive non-income	60.3	52.9	33.0	31.8	38.5	
Low income	30.7	39.0	54.1	59.6	51.9	
High income	9.1	6.1	7.8	3.2	5.4	
Religion, %						
Hindu	70.3	92.4	97.2	93.3	92.1	<0.01
Muslim	29.7	7.6	2.8	6.7	7.9	
Caste, %						
Scheduled caste	19.2	16.0	14.6	15.9	15.8	0.88
Scheduled tribe	3.3	5.0	6.2	3.0	4.2	
Other backwards caste	64.8	69.0	68.9	73.8	71.0	
General	12.7	10.0	10.4	7.4	9.1	
Marital status, %						
Single	10.6	8.1	3.5	6.5	6.4	0.28
Married	69.3	84.5	87.8	79.6	81.7	
Widow(er)	18.6	7.4	7.4	12.1	10.6	
Separated/divorced	1.5	0.0	1.2	1.8	1.3	
Housing quality, %						
Lowest level (*kuccha*)	62.0	53.9	57.0	46.1	51.6	0.25
Mixed (*semi-pucca*)	13.2	17.7	17.9	13.2	15.2	
Highest level (*pucca*)	24.8	28.3	25.1	40.7	33.2	
Owns land, %						
Yes	15.2	22.1	30.6	37.2	30.9	0.02
Depression symptom severity (total PHQ-9 score), %						
Moderate (10–14)	95.4	80.8	75.4	75.0	77.9	0.27
Moderately severe (15–19)	4.6	19.2	20.3	23.1	20.1	
Severe (⩾20)	0.0	0.0	4.3	1.9	2.0	
Survey round						
Round 1 (before MHCP implementation)	17.7	34.5	58.9	87.9	64.8	0.0001
Round 2 (after MHCP implementation)	82.3	65.5	41.1	12.1	35.2	

*p*-Values are calculated using χ^2^. Counts are unadjusted for sampling design; percentages are adjusted for sampling design.

The productive non-income group consisted of students and housewives.

### Objective 1: travel distance by survey round

Implementation of the MHCP reduced the median travel distance to a public depression treatment provider from 26.9 km in round 1 (25^th^ and 75^th^ percentiles: 16.0 km, 36.2 km; skewness 2.40) to 9.7 km in the second round (25^th^ and 75^th^ percentiles: 6.5 km, 16.8 km; skewness 4.29), (*p* < 0.0001).

### Objective 2: travel distance and treatment-seeking for depressive symptoms

As previously reported (Shidhaye *et al.*, 2019), of the 568 people with probable depression in both rounds, 75 (13.9%) sought treatment for these symptoms.

There was no evidence of an association between treatment-seeking and distance to a public depression treatment provider, either in unadjusted or adjusted models, with any provider or only formal providers (see Table 2). We checked for differences between rounds and found no evidence of an association at either time point (round 1: OR 1.00, 95% CI 0.99–1.01; round 2: OR 0.97, 95% CI 0.93–1.02).

**Table 2.** Travel distance to nearest public depression treatment provider and odds of seeking treatment for adults with probable depression (*n* = 568) in Sehore sub-district, Madhya Pradesh, India, 2013–2017

	Unadjusted OR (95% CI)	*p*	Adjusted OR (95% CI)	*p*
Use of any services for depression	1.01 (1.00–1.01)	0.16	1.00 (0.98–1.02)	0.78
Use of formal services for depression	1.00 (0.99–1.01)	0.73	0.99 (0.97–1.02)	0.69

Odds ratios, 95% CIs and *p*-values calculated using logistic regression.

Formal services include specialist doctors, generalist doctors, other mental health professionals (psychologists, counsellors and mental health nurses), other generalist health workers (social workers, community health workers, nurses, ANMs, ASHAs and AWWs) and case managers. Excludes ojha/guni/dev maharaj, traditional healers, herbalists, spiritualists or other providers.

Adjusted models include the following covariates: education level, marital status, symptom severity, gender, land ownership, employment, round, exposure to mental health communications, age group.

### Objective 3: treatment-seeking and travel distance among sub-groups

Table 3 shows the association between travel distance and treatment-seeking by the sub-group (where Wald *p*-values for interaction terms <0.10; full table in online Supplementary material). There was evidence of interaction with caste, employment status and perceived need for health care, weak evidence of interaction with age, but no evidence of any interaction effects by gender, education level, religion, marital status, housing type, land ownership, symptom severity or exposure to mental health communications.

**Table 3.** Sub-group analysis for distance to depression treatment provider and odds of treatment-seeking for adults with probable depression (*n* = 568) in Sehore sub-district, Madhya Pradesh, India, 2013–2017

	Adjusted OR (95% CI)	Stratum-specific *p*-value	Wald *p*-value for interaction terms
Caste			0.02
Scheduled castes	1.04 (1.01–1.06)	<0.01	
Scheduled tribes	0.98 (0.90–1.06)	0.54	
Other backward castes	0.98 (0.96–1.01)	0.15	
General castes	1.00 (0.97–1.04)	0.87	
Employment status			0.03
Unemployed	0.73 (0.60–0.90)	<0.01	
Productive no income	1.00 (0.98–1.02)	0.95	
Low income	1.01 (0.99–1.02)	0.59	
High income	0.98 (0.91–1.05)	0.55	
Perceived need for health care			0.02
Health care needed	0.99 (0.97–1.01)	0.32	
Health care not needed	1.02 (1.00–1.03)	0.06	

Odds ratios, *p*-values and confidence intervals were calculated with logistic regression.

Besides the interaction term, each model was adjusted for education level, marital status, symptom severity, gender, land ownership, employment, round, exposure to mental health communications and age group.

The effect sizes by caste and perceived need for health care were small and in the opposite direction from expected; e.g. for every 1 km increase in travel distance to the nearest treatment provider, individuals from scheduled castes had 4% higher odds of seeking treatment. There was a larger effect for the unemployed sub-group, with a 27% reduction in the odds of seeking treatment for every 1 km increase in travel distance.

## Discussion

### Principal findings

Travel distance to the nearest public depression treatment provider was significantly reduced after the implementation of the MHCP, but the proportion of people with probable depression who sought treatment remained low regardless of distance to services. To our knowledge, this is the first study from India to examine associations between travel distance and treatment-seeking for mental disorders.

The lack of evidence of an association between travel distance and treatment-seeking contrasts with literature from high-income countries (HIC) on ‘Jarvis’ law’ in mental health care (Bille, 1963; Davey and Giles, 1979; Stampfer *et al*., 1984; Shannon *et al*., 1986; Almog *et al*., 2004; Zulian *et al*., 2011; Packness *et al*., 2017). The narrow range of the confidence intervals indicates that this finding is unlikely to be due to low statistical power.

### Mechanisms and methodological differences

There are several potential explanations for the difference in findings compared to studies from HIC.

#### Threshold effects

Research from HIC has pointed to a ‘zone of indifference’, beyond which distance ceases to affect rates of mental health service use (Shannon *et al*., 1986; Stampfer *et al*., 1984). However, we found no evidence of such threshold effects in our data.

#### Over-utilisation

Distance decay effects in HIC primarily affect those with milder symptoms (Joseph and Boeckh, 1981) and may reflect over-utilisation of services by those in the vicinity of health services (Davey and Giles, 1979). Since the current analysis was restricted to people with probable depression, treatment-seeking by those without clinical need was largely excluded.

#### Population-based *v.* facility-based samples

Unlike most previous research on this topic, this study used a population-based sample. In facility-based studies, geographic differences in prevalence complicate the interpretation of utilisation data. Furthermore, it appears that the decision to seek *any* care may be influenced by different factors than the choice of provider among those who seek help (Fortney *et al*., 1998). In the Indian setting, where public services are not the only option, the location of public services may therefore influence the choice of provider but not the overall likelihood of treatment-seeking.

#### Differences in health systems

Perhaps most importantly, the context of medical pluralism in India means that, unlike countries with more homogeneous health systems, access to services is far more complex than the availability or accessibility of public, allopathic health services (Halliburton, 2004). The current findings clearly undermine the assumption that reducing travel distance to public mental health services will reduce the overall treatment gap for depression in India and comparable settings, and suggest that future research should focus on the role of the wider health sector in influencing treatment-seeking for depression, including private and complementary providers (Quack, 2012).

### Implications

Both international and Indian mental health policies advocate the integration of mental health services into primary care (WHO, 2008; WHO & WONCA, 2008; Ministry of Health & Family Welfare, 2014), partly on the basis that this improves the geographic accessibility of services. However, this study found that those living in the immediate vicinity of public mental health services are no more likely to seek care than those facing longer journeys, demonstrating that distance to the nearest public depression service is not a primary factor in explaining low treatment-seeking rates.

One interpretation of this finding is that increased accessibility of services is insufficient to reduce the treatment gap in areas where demand for these services is very low. The Vidarbha Stress and Health Program, another depression programme in central India, included village-level demand generation activities and reported a six-fold increase in treatment-seeking in the implementation area (Shidhaye *et al*., 2017), in contrast to PRIME.

An alternative interpretation is that the accessibility of *public, allopathic* mental health services is of little relevance to treatment-seeking behaviour, since these represent a minority of services and are not the preferred choice of healthcare for many (De Costa and Diwan, 2007; De Costa and Johannson, 2011). The finding that the location of public, allopathic mental health services has negligible effects on the treatment gap in this context should prompt us to re-examine the sole focus on these in current mental health policy, and to take into account the complexity of the health system in future initiatives to improve access to care (Dalal, 2005).

Caution is needed in interpreting the results of the sub-group analyses, since multiple tests were performed, increasing the likelihood of chance findings, and the effect sizes were small. The preliminary finding that for some groups, those living further from public health services were slightly *more* likely to seek treatment, could indicate that other mechanisms, such as stigmatisation, might be involved. Further investigation and replication of these results are necessary to understand the processes involved in decisions around treatment-seeking for depression. Unemployed adults may be more sensitive to travel distance to public services as an obstacle to treatment-seeking than the rest of the population, although this group represents only 4.2% of individuals with probable depression.

### Strengths

This study used a community-based sample, and therefore reflects the general population better than facility-based studies. The sample size compares favourably with previous international (Roberts *et al*., 2018) and India-based studies of treatment-seeking for mental disorders (Lahariya *et al*., 2010; Mishra *et al*., 2011; Jain *et al*., 2012; Andrade *et al*., 2014; Mathias *et al*., 2015; Shidhaye *et al*., 2016*a*). We chose network analysis measures as a rigorous method of calculating travel distance (Fortney *et al*., 2000; Apparicio *et al*., 2008; Nesbitt *et al*., 2014).

### Limitations

GPS coordinates were missing for some households, including a large proportion in round 1. However, we believe that the error introduced by substituting village centroid coordinates in these cases is relatively small, given the size of villages in the implementation area. When we excluded those with missing coordinates, we found no difference in our results for either round.

Our exposure of interest was an estimate of travel distance, but we lacked data on access to transportation to convert these to travel time estimates, which may be of greater relevance to treatment-seeking decisions. Future research should generate more nuanced estimates of travel time and cost for this setting.

The data were cross-sectional, meaning that some factors (e.g. symptom severity, perceived needs) may have changed over the period asked about. Recall bias is possible since we used self-reported outcome data (Bhandari and Wagner, 2006), although this affects binary measures of treatment-seeking less than measures of volume or frequency (Raina *et al*., 2002; Carroll *et al*., 2016). Differential misclassification is possible if longer journeys lead to greater recollection of treatment-seeking, which could explain the apparent positive association between distance and treatment-seeking among some groups.

We did not have data on the location of private or complementary providers in order to calculate travel distance to these, since these are highly numerous and no official register of these services exists. Future studies might usefully attempt to map these to generate more context-specific measures of geographic accessibility.

It would also have been useful to investigate whether distance to the nearest public provider affected the likelihood of consulting a public rather than private or complementary provider, among those who sought depression treatment. However, the number who sought treatment was too small to enable this. Finally, the low rate of treatment-seeking overall limits the chances of finding an association between distance and treatment-seeking, although the narrow confidence intervals suggest a relatively high degree of precision in our estimates.

## Conclusion

The current study identified no association between travel distance to the nearest public depression treatment provider and treatment-seeking for probable depression, except for the small sub-group of unemployed adults. Low geographic accessibility of public, allopathic services does not explain the treatment gap for depression in this context, and decentralising public mental services to reduce travel distance will not of itself reduce the treatment gap for depression in rural India. Future research should examine alternative measures of geographic accessibility of mental health services, taking into account the health systems context of India which includes many private and complementary service providers.

## Supplementary material

For supplementary material accompanying this paper visit http://dx.doi.org/10.1017/S204579601900088X. click here to view supplementary material

## Data

Interested parties may notify the PRIME investigators of their interest in collaboration, including access to the data set analysed here, through the following website: http://www.prime.uct.ac.za/contact-prime.

## References

[ref1] Aday LA and Andersen R (1974) A framework for the study of access to medical care. Health Services Research 9, 208. 4436074 PMC1071804

[ref2] Ager A and Pepper K (2005) Patterns of health service utilization and perceptions of needs and services in rural Orissa. Health Policy and Planning 20, 176–184. 15840633 10.1093/heapol/czi021

[ref3] Albert S, Nongrum M, Webb EL, Porter JDH and Kharkongor GC (2015) Medical pluralism among indigenous peoples in northeast India-implications for health policy. Tropical Medicine & International Health 20, 952–960. 25753562 10.1111/tmi.12499

[ref4] Almog M, Curtis S, Copeland A and Congdon P (2004) Geographical variation in acute psychiatric admissions within New York city 1990–2000: growing inequalities in service use? Social Science & Medicine 59, 361–376. 15110426 10.1016/j.socscimed.2003.10.019

[ref5] Andrade LH, Alonso J, Mneimneh Z, Wells J, Al-Hamzawi A, Borges G, Bromet E, Bruffaerts R, De Girolamo G and De Graaf R (2014) Barriers to mental health treatment: results from the WHO World Mental Health surveys. Psychological Medicine 44, 1303–1317. 23931656 10.1017/S0033291713001943 PMC4100460

[ref6] Apparicio P, Abdelmajid M, Riva M and Shearmur R (2008) Comparing alternative approaches to measuring the geographical accessibility of urban health services: distance types and aggregation-error issues. International Journal of Health Geographics 7, 7. 18282284 10.1186/1476-072X-7-7 PMC2265683

[ref8] Bhandari A and Wagner T (2006) Self-reported utilization of health care services: improving measurement and accuracy. Medical Care Research and Review 63, 217–235. 16595412 10.1177/1077558705285298

[ref9] Bille M (1963) The influence of distance of admissions to mental hospitals: first admissions. Acta Psychiatrica Scandinavica 38, 226–233. 14085197 10.1111/j.1600-0447.1963.tb07869.x

[ref10] Carroll M, Sutherland G, Kemp-Casey A and Kinner SA (2016) Agreement between self-reported healthcare service use and administrative records in a longitudinal study of adults recently released from prison. Health & Justice 4, 11. 27942429 10.1186/s40352-016-0042-x PMC5121169

[ref12] Dalal AK (2005) Integrating traditional services within primary health care. Journal of Health Management 7, 249–262.

[ref13] Davey S and Giles G (1979) Spatial factors in mental health care in Tasmania. Social Science & Medicine. Part D: Medical Geography 13, 87–94. 10.1016/0160-8002(79)90055-8 494013

[ref15] De Costa A and Diwan V (2007) ‘Where is the public health sector?’: Public and private sector healthcare provision in Madhya Pradesh, India. Health Policy 84, 269–276. 17540472 10.1016/j.healthpol.2007.04.004

[ref16] De Costa A and Johannson E (2011) By ‘default or design’? The expansion of the private health care sector in Madhya Pradesh, India. Health Policy 103, 283–289. 21782268 10.1016/j.healthpol.2011.06.005

[ref17] Delamater PL, Messina JP, Shortridge AM and Grady SC (2012) Measuring geographic access to health care: raster and network-based methods. International Journal of Health Geographics 11, 15. 22587023 10.1186/1476-072X-11-15 PMC3511293

[ref18] De Silva MJ, Lee L, Fuhr DC, Rathod S, Chisholm D, Schellenberg J and Patel V (2014) Estimating the coverage of mental health programmes: a systematic review. International Journal of Epidemiology 43, 341–353. 24760874 10.1093/ije/dyt191 PMC3997372

[ref19] De Silva MJ, Rathod SD, Hanlon C, Breuer E, Chisholm D, Fekadu A, Jordans M, Kigozi F, Petersen I, Shidhaye R, Medhin G, Ssebunnya J, Prince M, Thornicroft G, Tomlinson M, Lund C and Patel V (2016) Evaluation of district mental healthcare plans: the PRIME consortium methodology. British Journal of Psychiatry 208(Suppl 56), s63–s70. 26447175 10.1192/bjp.bp.114.153858 PMC4698558

[ref20] Ecks S and Basu S (2009) The unlicensed lives of antidepressants in India: generic drugs, unqualified practitioners, and floating prescriptions. Transcultural Psychiatry 46, 86–106. 19293281 10.1177/1363461509102289

[ref21] Ecks S and Basu S (2014) ‘We always live in fear’: antidepressant prescriptions by unlicensed doctors in India. Culture, Medicine, and Psychiatry 38, 197–216. 10.1007/s11013-014-9368-9 24705978

[ref22] Esri (2011) ArcGIS Desktop: Release 10. Redlands, CA: Environmental Systems Research Institute.

[ref23] Fortney J, Rost K and Zhang M (1998) A joint choice model of the decision to seek depression treatment and choice of provider sector. Medical Care 36, 307–320. 9520956 10.1097/00005650-199803000-00008

[ref25] Fortney J, Rost K and Warren J (2000) Comparing alternative methods of measuring geographic access to health services. Health Services and Outcomes Research Methodology 1, 173–184.

[ref26] Gururaj G, Varghese M, Benegal V, Rao G, Pathak K, Singh L and Misra R (2016) National Mental Health Survey of India, 2015–16: Summary. Bengaluru: National Institute of Mental Health and Neuro Sciences, NIMHANS Publication.

[ref27] Halliburton M (2004) Finding a fit: psychiatric pluralism in South India and its implications for WHO studies of mental disorder. Transcultural Psychiatry 41, 80–98. 15171208 10.1177/1363461504041355

[ref28] Hanlon C, Luitel NP, Kathree T, Murhar V, Shrivasta S, Medhin G, Ssebunnya J, Fekadu A, Shidhaye R and Petersen I (2014) Challenges and opportunities for implementing integrated mental health care: a district level situation analysis from five low-and middle-income countries. PLoS One 9, e88437. 24558389 10.1371/journal.pone.0088437 PMC3928234

[ref31] Hunter JM and Shannon GW (1985) Jarvis revisited: distance decay in service areas of mid-19th century asylums. The Professional Geographer 37, 296–302. 11617962 10.1111/j.0033-0124.1985.00296.x

[ref32] Jain N, Gautam S, Jain S, Gupta I, Batra L, Sharma R and Singh H (2012) Pathway to psychiatric care in a tertiary mental health facility in Jaipur, India. Asian Journal of Psychiatry 5, 303–308. 23174437 10.1016/j.ajp.2012.04.003

[ref33] Joseph AE and Boeckh JL (1981) Locational variation in mental health care utilization dependent upon diagnosis: a Canadian example. Social Science & Medicine. Part D: Medical Geography 15, 395–404. 10.1016/0160-8002(81)90058-7 7323872

[ref34] Kohn R, Saxena S, Levav I and Saraceno B (2004) The treatment gap in mental health care. Bulletin of the World Health Organization 82, 858–866. 15640922 PMC2623050

[ref36] Kumar R, Singh M and Kaur M (1997) Impact of health centre availability on utilisation of maternity care and pregnancy outcome in a rural area of Haryana. Journal of the Indian Medical Association 95, 448–450. 9492451

[ref37] Kumar AKS, Chen LC, Choudhury M, Ganju S, Mahajan V, Sinha A and Sen A (2011) Financing health care for all: challenges and opportunities. Lancet 377, 668–679. 21227490 10.1016/S0140-6736(10)61884-3

[ref38] Kumar S, Dansereau EA and Murray CJ (2014) Does distance matter for institutional delivery in rural India? Applied Economics 46, 4091–4103.

[ref39] Lahariya C, Singhal S, Gupta S and Mishra A (2010) Pathway of care among psychiatric patients attending a mental health institution in central India. Indian Journal of Psychiatry 52, 333. 21267367 10.4103/0019-5545.74308 PMC3025159

[ref41] Lund C, Tomlinson M, De Silva M, Fekadu A, Shidhaye R, Jordans M, Petersen I, Bhana A, Kigozi F, Prince M, Thornicroft G, Hanlon C, Kakuma R, McDaid D, Saxena S, Chisholm D, Raja S, Kippen-Wood S, Honikman S, Fairall L and Patel V (2012) PRIME: a programme to reduce the treatment gap for mental disorders in five low- and middle-income countries. PLoS Medicine 9, e1001359. 23300387 10.1371/journal.pmed.1001359 PMC3531506

[ref42] Manea L, Gilbody S and McMillan D (2012) Optimal cut-off score for diagnosing depression with the Patient Health Questionnaire (PHQ-9): a meta-analysis. Canadian Medical Association Journal 184, E191–E196. 22184363 10.1503/cmaj.110829 PMC3281183

[ref43] Manea L, Gilbody S and McMillan D (2015) A diagnostic meta-analysis of the Patient Health Questionnaire-9 (PHQ-9) algorithm scoring method as a screen for depression. General Hospital Psychiatry 37, 67–75. 25439733 10.1016/j.genhosppsych.2014.09.009

[ref44] Mathias K, Goicolea I, Kermode M, Singh L, Shidhaye R and San Sebastian M (2015) Cross-sectional study of depression and help-seeking in Uttarakhand, North India. BMJ Open 5, e008992. 10.1136/bmjopen-2015-008992 PMC4663438 26589428

[ref45] Ministry of Health & Family Welfare, Government of India (2014) New Pathways. New Hope: National Mental Health Policy of India.

[ref46] Mishra N, Nagpal SS, Chadda RK and Sood M (2011) Help-seeking behavior of patients with mental health problems visiting a tertiary care center in north India. Indian Journal of Psychiatry 53, 234. 22135442 10.4103/0019-5545.86814 PMC3221180

[ref47] Mizushima Laboratory (2013) India Place Finder, Department of Oriental History, Graduate School of Humanities and Sociology, The University of Tokyo. Available at http://india.csis.u-tokyo.ac.jp/ (Accessed 5 January 2019).

[ref48] Nesbitt RC, Gabrysch S, Laub A, Soremekun S, Manu A, Kirkwood BR, Amenga-Etego S, Wiru K, Höfle B and Grundy C (2014) Methods to measure potential spatial access to delivery care in low-and middle-income countries: a case study in rural Ghana. International Journal of Health Geographics 13, 25–25. 24964931 10.1186/1476-072X-13-25 PMC4086697

[ref49] Office of the Registrar General and Census Commissioner, I. Census (2011) Available at http://www.censusindia.gov.in/ (Accessed 12 February 2019).

[ref53] Packness A, Waldorff FB, dePont Christensen R, Hastrup LH, Simonsen E, Vestergaard M and Halling A (2017) Impact of socioeconomic position and distance on mental health care utilization: a nationwide Danish follow-up study. Social Psychiatry and Psychiatric Epidemiology 52, 1405–1413. 28849245 10.1007/s00127-017-1437-2 PMC5663810

[ref55] Penchansky R and Thomas JW (1981) The concept of access: definition and relationship to consumer satisfaction. Medical Care 19. 10.1097/00005650-198102000-00001 7206846

[ref56] Peters DH, Garg A, Bloom G, Walker DG, Brieger WR and Rahman MH (2008) Poverty and access to health care in developing countries. Annals of the New York Academy of Sciences 1136, 161–171. 17954679 10.1196/annals.1425.011

[ref57] Quack J (2012) Ignorance and utilization: mental health care outside the purview of the Indian state. Anthropology & Medicine 19, 277–290. 22870866 10.1080/13648470.2012.692357

[ref58] Raina P, Torrance-Rynard V, Wong M and Woodward C (2002) Agreement between self-reported and routinely collected health-care utilization data among seniors. Health Services Research 37, 751–774. 12132604 10.1111/1475-6773.00047 PMC1434660

[ref59] Ranga V and Panda P (2016) Private non-degree practitioners and spatial access to out-patient care in rural India. GeoJournal 81, 267–280.

[ref60] Rathod SD, De Silva MJ, Ssebunnya J, Breuer E, Murhar V, Luitel NP, Medhin G, Kigozi F, Shidhaye R and Fekadu A (2016) Treatment contact coverage for probable depressive and probable alcohol use disorders in four low-and middle-income country districts: the PRIME cross-sectional community surveys. PLoS One 11, e0162038. 27632166 10.1371/journal.pone.0162038 PMC5025033

[ref62] Roberts T, Miguel Esponda G, Krupchanka D, Shidhaye R, Patel V and Rathod S (2018) Factors associated with health service utilisation for common mental disorders: a systematic review. BMC Psychiatry 18, 262. 30134869 10.1186/s12888-018-1837-1 PMC6104009

[ref63] Sawhney N (1993) Management of family welfare programme in Uttar Pradesh. Infrastructure utilization quality of services supervision and MIS In Premi MK (ed.), Family Planning and MCH in Uttar Pradesh. New Delhi, India: Indian Association for the Study of Population, pp. 50–67.

[ref65] Selvaraj S and Karan AK (2009) Deepening health insecurity in India: evidence from national sample surveys since 1980s. Economic and Political Weekly, 55–60.

[ref66] Shankar D (2015) Health sector reforms for 21st century healthcare. Journal of Ayurveda and Integrative Medicine 6, 4. 25878456 10.4103/0975-9476.154214 PMC4395927

[ref67] Shannon GW, Bashshur RL and Lovett JE (1986) Distance and the use of mental health services. The Milbank Quarterly, 302–330. 3086685

[ref68] Shariff A and Singh G (2002) Determinants of maternal health care utilisation in India: Evidence from a recent household survey. National Council of Applied Economic Research. New Delhi, India. Working Paper Series 85. Available at https://core.ac.uk/download/pdf/6356570.pdf (Accessed 25 August 2018).

[ref69] Shidhaye R, Raja A, Shrivastava S, Murhar V, Ramaswamy R and Patel V (2015) Challenges for transformation: a situational analysis of mental health care services in Sehore District, Madhya Pradesh. Community Mental Health Journal 51, 903–912. 26059181 10.1007/s10597-015-9893-1 PMC4615668

[ref70] Shidhaye R, Gangale S and Patel V (2016a) Prevalence and treatment coverage for depression: a population-based survey in Vidarbha, India. Social Psychiatry and Psychiatric Epidemiology 51, 993–1003. 27106852 10.1007/s00127-016-1220-9 PMC4947473

[ref71] Shidhaye R, Shrivastava S, Murhar V, Samudre S, Ahuja S, Ramaswamy R and Patel V (2016b) Development and piloting of a plan for integrating mental health in primary care in Sehore district, Madhya Pradesh, India. British Journal of Psychiatry 208(Suppl 56), s13–s20. 26447172 10.1192/bjp.bp.114.153700 PMC4698552

[ref72] Shidhaye R, Murhar V, Gangale S, Aldridge L, Shastri R, Parikh R, Shrivastava R, Damle S, Raja T, Nadkarni A and Patel V (2017) The effect of VISHRAM, a grass-roots community-based mental health programme, on the treatment gap for depression in rural communities in India: a population-based study. The Lancet Psychiatry 4, 128–135. 28063879 10.1016/S2215-0366(16)30424-2

[ref74] Shidhaye R, Baron E, Murhar V, Rathod S, Khan A, Singh A, Shrivastava S, Muke S, Shrivastava R and Lund C (2019) Community, facility and individual level impact of integrating mental health screening and treatment into the primary healthcare system in Sehore district, Madhya Pradesh, India. BMJ Global Health 4, e001344. 10.1136/bmjgh-2018-001344 PMC6528752 31179034

[ref75] Stampfer H, Reymond J, Burvill P and Carlson J (1984) The relationship between distance from inpatient facilities and the rate of psychiatric admissions in Western Australia. Social Science & Medicine 19, 879–884. 6505754 10.1016/0277-9536(84)90406-4

[ref76] StataCorp (2015) Stata Statistical Software: Release 14. College Station, TX: StataCorp LP.

[ref81] Vissandjée B, Barlow R and Fraser D (1997) Utilization of health services among rural women in Gujarat, India. Public Health 111, 135–148. 9175456 10.1016/s0033-3506(97)00572-6

[ref82] WHO (2008) mhGAP: Mental Health Gap Action Programme. Scaling up care for mental, neurological and substance use disorders. World Health Organization. Available at https://apps.who.int/iris/bitstream/handle/10665/43809/9789241596206_eng.pdf (Accessed 17 October 2018). 26290926

[ref83] WHO (2017) Depression and Other Common Mental Disorders: Global Health Estimates. Geneva. Available at https://apps.who.int/iris/bitstream/handle/10665/254610/WHO-MSD-MER-2017.2-eng.pdf (Accessed 16 October 2018).

[ref84] WHO & WONCA (2008) Integrating mental health into primary care: A global perspective. World Health Organization and World Organization of Family Doctors. Available at https://apps.who.int/iris/bitstream/handle/10665/43935/9789241563680_eng.pdf (Accessed 17 October 2018).

[ref85] Wilcox CE, Washburn R and Patel V (2007) Seeking help for attention deficit hyperactivity disorder in developing countries: a study of parental explanatory models in Goa, India. Social Science & Medicine 64, 1600–1610. 17267087 10.1016/j.socscimed.2006.11.032

[ref88] Zulian G, Donisi V, Secco G, Pertile R, Tansella M and Amaddeo F (2011) How are caseload and service utilisation of psychiatric services influenced by distance? A geographical approach to the study of community-based mental health services. Social Psychiatry and Psychiatric Epidemiology 46, 881–891. 20577712 10.1007/s00127-010-0257-4

