# Supplementary material for: Distance to health services and treatment-seeking for depressive symptoms in rural India: a repeated cross-sectional study
Source: Epidemiol Psychiatr Sci. 2020 Jan 13;29:e92. doi: 10.1017/S204579601900088X (PMC7214702; doi:10.1017/S204579601900088X)
Supplement: Supplementary file 1 [file S204579601900088Xsup001.docx]

## Supplementary material

**Table 4.** Sub-group analysis for distance to depression treatment provider and odds of treatment-seeking for adults with probable depression (n=568) in Sehore sub-district, Madhya Pradesh, India, 2013-2017.

|  | **Adjusted OR (95% CI)** | **Stratum-specific P-value** | **Wald P-value for interaction terms** |
| --- | --- | --- | --- |
| *Caste* | | | 0.02 |
| Scheduled castes | 1.04 (1.01-1.06) | <0.01 |  |
| Scheduled tribes | 0.98 (0.90-1.06) | 0.54 |  |
| Other backward castes | 0.98 (0.96-1.01) | 0.15 |  |
| General castes | 1.00 (0.97-1.04) | 0.87 |  |
| *Employment status* | | | 0.03 |
| Unemployed | 0.73 (0.60-0.90) | <0.01 |  |
| Productive no income | 1.00 (0.98-1.02) | 0.95 |  |
| Low income | 1.01 (0.99-1.02) | 0.59 |  |
| High income | 0.98 (0.91-1.05) | 0.55 |  |
| *Educational attainment* | | | 0.62 |
| Less than primary | 1.00 (0.99-1.02) | 0.70 |  |
| Primary or more | 0.99 (0.94-1.04) | 0.70 |  |
| *Perceived need for health care* | | | 0.02 |
| Health care needed | 0.99 (0.97-1.01) | 0.32 |  |
| Health care not needed | 1.02 (1.00-1.03) | 0.06 |  |
| *Housing quality* | | | 0.25 |
| Lowest level (*kuccha*) | 1.01 (0.99-1.02) | 0.32 |  |
| Mixed (*semi-pucca*) | 0.97 (0.92-1.02) | 0.26 |  |
| Highest level (*pucca*) | 0.99 (0.97-1.02) | 0.72 |  |
| *Gender* | | | 0.54 |
| Male | 1.00 (0.96-1.03) | 0.82 |  |
| Female | 1.01 (0.99-1.02) | 0.37 |  |
| *Owns land* | | | 0.49 |
| Yes | 1.00 (0.97-1.03) | 0.80 |  |
| No | 1.01 (0.99-1.02) | 0.51 |  |
| *Symptom severity (total PHQ-9 score)* | | | 0.48 |
| Moderate (10-14) | 1.00 (0.98-1.02) | 0.98 |  |
| Moderately severe  (15-19) | 1.01 (0.98-1.04) | 0.60 |  |
| Severe (≥20) | 0.97 (0.91-1.03) | 0.33 |  |

*Odds ratios, P-values and confidence intervals were calculated with logistic regression. Besides the interaction term, each model was adjusted for education level, marital status, symptom severity, gender, land ownership, employment, survey round, exposure to mental health communications, and age group.*
